# 3D Virtual Reality Performance Metrics as a Future Fatigue Biomarker in Myalgic Encephalomyelitis/Chronic Fatigue Syndrome (ME/CFS)

**DOI:** 10.3390/biomedicines14040855

**Published:** 2026-04-09

**Authors:** Anja-Maria Ladek, Leonie Priebe, Thomas Harrer, Ellen Harrer, Georg Michelson, Thomas S. Knauer, Diogo X. Dias-Nunes, Christian Y. Mardin, Antonio Bergua, Bettina Hohberger

**Affiliations:** 1 Department of Ophthalmology, Universitätsklinikum Erlangen, Friedrich-Alexander-Universität Erlangen-Nürnberg, 91054 Erlangen, Germany; 2 PhD Medical Sciences, Department of Human Medicine, Paracelsus Medical University, 5020 Salzburg, Austria; 3 Department of Internal Medicine 3-Rheumatology and Immunology, Universitätsklinikum Erlangen, Friedrich-Alexander-Universität Erlangen-Nürnberg, 91054 Erlangen, Germany; 4 Deutsches Zentrum Immuntherapie and Center for Rare Diseases Erlangen, Universitätsklinikum Erlangen, Friedrich-Alexander-Universität Erlangen-Nürnberg, 91054 Erlangen, Germany; 5 Infectious Diseases and Immunodeficiency Section, Department of Medicine 3, Universitätsklinikum Erlangen, Friedrich-Alexander-Universität Erlangen-Nürnberg, 91054 Erlangen, Germany

**Keywords:** ME/CFS, post-exertional malaise, PEM, reaction time, 3-dimensional, Post-COVID, fatigue

## Abstract

**Background:** Myalgic encephalomyelitis/chronic fatigue syndrome (ME/CFS) is a debilitating disorder, characterized by symptoms such as post-exertional malaise (PEM) and cognitive impairments. This study assessed reaction time (RT) metrics in three-dimensional (3D) visual tasks with the aim of objectively quantifying the cognitive impairments in ME/CFS patients compared to controls. **Methods:** A total of 120 participants (60 ME/CFS patients and 60 controls) were recruited at the Department of Ophthalmology, Universität of Erlangen-Nürnberg. RT was assessed using a virtual reality–oculomotor test system, presenting 3D stimuli at three disparity levels (275″, 550″, and 1100″) within three gaming repetitions (R1, R2, and R3). Mixed-effects models were used to evaluate group differences, with age and gender as covariates. Pairwise contrasts were calculated to assess changes across repetitions. Fatigue self-assessments were recorded by validated questionnaires, (FACIT Fatigue Scale, Chalder Fatigue Scale, Bell Score and Health Assessment Questionnaire), and their correlation with RT metrics was portrayed using a Spearman correlation matrix. **Results:** Estimated means (EM-means) for RT were significantly prolonged in ME/CFS patients compared to controls at disparity 275″ (1969 ms vs. 1384 ms; *p* = 0.0001), 550″ (1409 vs. 1071 ms; *p* = 0.0012) and 1100″ (1126 ms vs. 891 ms; *p* = 0.00223). Age was a significant covariate (*p* < 0.001), while gender showed no effect. Both groups demonstrated improvements in RT over repetitions; however, ME/CFS patients showed a significantly lower improvement compared to controls, reaching significance in R3 (*p* = 0.0042). RT metrics did not correlate with patients’ self-assessment scores. **Conclusions:** ME/CFS patients showed consistently slower RTs compared to controls, particularly in later, easier gaming repetitions, potentially reflecting the impact of fatigue.

## 1. Introduction

Myalgic encephalomyelitis/chronic fatigue syndrome (ME/CFS) is a debilitating disorder, often caused post-infectiously. Several pathophysiologic mechanisms for ME/CFS including impaired perfusion by vascular dysfunction, altered biomechanics of erythrocytes, autoantibodies against G-protein coupled receptors, altered metabolism by mitochondrial dysfunction and impaired neuronal signal transduction have been reported [1,2,3,4,5,6]. This variability of proposed pathomechanisms likely hints at disease subtypes, and respective biomarkers including markers of mitochondrial dysfunction, immunological alterations, viral reactivations, neuroendocrine disturbances and genetic factors were developed [7,8].

ME/CFS is characterized by its cardinal symptom of post-exertional malaise (PEM), accompanied by physical and mental fatigue, brain fog as well as immunological, autonomic and/or endocrine dysfunction [7]. A global prevalence of 0.89% [9] is likely underreported, as the disease is not universally defined and accepted worldwide. According to the international consensus criteria [10], a primary obligatory symptom is exertion-induced rapid and increased fatigability with prolonged recovery. Additionally, three symptoms out of four neurological impairment categories must be present, among others (e.g., neurological impairments, pain, headache, and unrefreshed sleep) [10]. Currently, the diagnosis of ME/CFS is based on the consensus criteria questionnaire and the absence of other causal disorders (e.g., Hashimoto thyroiditis). Post-COVID syndrome (PCS) and ME/CFS show overlapping symptoms; thus, a subgroup of PCS patients also conform with the ME/CFS diagnostic criteria [11]. The two diagnoses are however often conflated together. American health record analysis showed a significant overlap of symptoms and diagnosis [12]. Among the common overlap, concentration difficulties and fatigue were the most common, while cardiac and respiratory conditions were more commonly associated with PCS, and pain, sleep disturbances and neuropsychiatric symptoms with ME/CFS, respectively [12].

Disease severity and symptomatic treatment effects are most commonly assessed with various fatigue questionnaires, lacking objective biomarkers in daily routine. Further, patients may struggle objectively reflecting their symptom severity in the context of non-personal questions [13]. This problem is highly relevant, as the absence of tangible objective parameters leads physicians to under- and misdiagnose the condition [14], and treatment effects, particularly partial improvements, are difficult to track.

Recently, several stereoptic performance and neurovisual coordination tools based on VR have been developed to diagnose or track neurological conditions [15,16,17,18,19]. Two-dimensional (2D) and three-dimensional (3D) tasks elicit different cortical activation patterns [20], and 3D stimuli have been shown to impose greater cognitive loads [21]. Among these 3D VR test systems, the virtual reality–oculomotor test system (VR-OTS) has been established in the diagnostics of sports-related head trauma [22]. The device is a licensed medical device, testing reaction time (RT) in a 3D-stereoptic game at varying difficulties and visual field locations. It was previously validated and used for the assessment of PCS patients [23,24], showing a significantly increased RT in the PCS group at all tested difficulties [23]. The task is simple and easy to explain. As it requires focus, reaction speed and depth perception, all among the international diagnostic criteria of ME/CFS, we hypothesized that VR-OTS may be a diagnostic or functional assessment tool for ME/CFS and would possibly reflect self-assessment scores. Thus, it was the aim of the present study to investigate RT in a 3D VR in patients with ME/FS compared to controls and its change over test duration.

## 2. Materials and Methods

### 2.1. Study Population

A total of 120 participants were recruited at the Department of Ophthalmology, Universitätsklinikum Erlangen, Friedrich-Alexander Universität Erlangen-Nürnberg: 60 patients with ME/CFS (33 female, 27 male) and 60 healthy controls (30 female, 30 male). Patients in the ME/CFS group fulfilled the Canadian Diagnostic Criteria for ME/CFS at the time of study participation and had a formal diagnosis from an independent physician with symptoms occurring prior to 2020 or with clear records of COVID-19 being ruled out during the initial infection. Participants responded to a study call posted across various networks within the university and across self-help channels and were not recruited directly from in- and outpatient ophthalmologic departments. Those with a history of amblyopia, strabismus or nystagmus were excluded. The study has been reviewed and approved by the ethics committee of the Friedrich-Alexander Universität Erlangen-Nürnberg (295_20_B) and was performed in accordance with the tenets of the Declaration of Helsinki. Every participant provided written informed consent prior to enrollment.

### 2.2. Questionnaires

Participants were asked to fill out self-assessment questionnaires and binary anamnestic questions targeting fatigue on the same day of study participation. Self-assessment questionnaires included: Bell Score, which scores the severity of chronic fatigue syndrome according to the possible activity level and the frequency of symptoms between 0 (worst) and 100 (best) [25], and the Chalder Fatigue Scale, assessing psychological and physiological fatigue; a total score of 3 or less represents non-fatigued individuals, with 11 being the highest score [26]. Further, binary (yes/no) amnestic questions were asked, whether brain fog, fatigue or impaired concentration are chronically experienced. Controls who answered yes to these binary anamnestic questions were excluded from the study.

Additionally, ME/CFS patients had to satisfy the Canadian Criteria for Myalgic Encephalomyelitis/Chronic Fatigue Syndrome, the diagnostic questionnaire referenced by the international consensus criteria [10], and were asked to fill out two further questionnaires: the Health Assessment Questionnaire Disability Index (HAQ-DI) [27], which scores difficulties performing simple daily tasks between 0 (no disability) and 3 (severe disability) and accounts for the use of accessibility instruments or other’s help, and the FACIT Fatigue Scale, which asks the patient to judge the severity of psychological or physical fatigue symptoms during the past 7 days and is scored between 0 (worst) and 52 (best) [28].

### 2.3. VR-OTS—Virtual Reality–Oculomotor Test System

**Figure 1:**
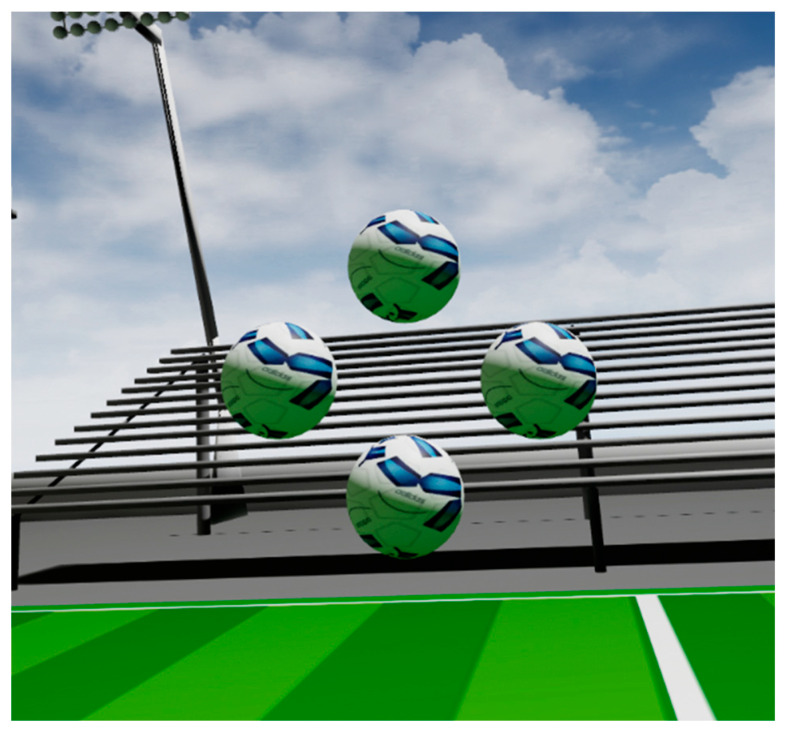
A screenshot from the VR application showing the four balls of a stimulus within the arena. The ball formation appears static to the visual field, e.g., a central stimulus remains central even when turning the head or the eyes. During the assessment, this is perceived in 3D using the VR-OTS headset.

The test setup (VR-OTS, Talking Eyes, Erlangen, Germany), protocol and hard- and software used in our group have been explained previously [23,24]. No formal simulator sickness assessment was performed. Participants were instructed to immediately report any discomfort. No premature test termination occurred. In brief, four balls were presented to the participants at either of nine locations: central position and 8 peripheral positions (4 diagonal and 4 straight peripheral positions). Figure 1 shows a screenshot of the game environment and the stimuli. Once the assessment starts, one of the four balls appears closer than the others at three different difficulty levels. In a stereoscopic setting, the angle between an object and the two eyes is defined as disparity. The difference of disparities when comparing two objects represents the difficulty level. The smaller the disparity, the harder it is to distinguish the differences between the objects. Disparities were 275, 550 and 1100 arcseconds (″) during the test, with 275″ being the smallest disparity difference displayable by the hardware. All disparities were displayed randomly at each of the 9 directions three times per gaming round, resulting in 81 stimuli for each single assessment. The participant was asked to select the closer ball as fast as possible (i.e., reaction time, RT). The test was performed three times per participant. Only correct answers were analyzed. RT, RT-slope, GAIN1, and GAIN2 were used for analysis. RT-slope refers to the slope of RT with easier difficulty in a given round. GAIN1 and GAIN2 represent the increase in RT from one disparity to the more difficult one, from 550″ to 275″ and 1100″ to 550″, respectively. None of the participants had any prior experience with the test system. In contrast to our previous study in PCS patients [23,24], all three rounds were analyzed, as a chief symptom of ME/CFS patients is mental fatigue or brain fog resulting in cognitive difficulties. We wanted to gain insight into the change in reaction time across rounds, in order to possibly detect potential differences in learning speed and fatigue which might represent brain fog and cognitive difficulties, two highly prevalent complaints in ME/CFS.

### 2.4. Statistical Analysis

Variables were checked for normalcy and standardized where necessary. To evaluate differences between the control and MECFS group across several variables (275″, 550″, 1100″, RT-slope, GAIN1, and GAIN2), a series of mixed-effects models were conducted. All three measurements were included as repeated measures in the models to account for within-subject variability. Age and gender were included as covariates to control for their potential confounding effects. For each variable, the group effect (control vs. ME/CFS) and the effect of gaming round were assessed. The results were reported with estimates, standard errors (SEs), *t*-values, *p*-values, and 95% confidence intervals (CIs). Estimated marginal means (EM-means) for each group were also calculated for clarity. EM-means represent the adjusted group means derived from the mixed-effects model. These means account for the effects of covariates (age and gender in this study) by estimating what the group averages would be if all participants had the same values for these covariates. EM-means provide a clearer comparison between groups (control vs. ME/CFS) by controlling for variability due to these additional factors. To further explore the differences, pairwise contrasts were calculated for the group variable to determine which comparisons were statistically significant (across the 3 rounds). These contrasts were assessed using post hoc tests (Tukey test). These adjustments were made to get more accurate *p*-values for each pairwise comparison and thus avoid inflating the Type I error rate. The pairwise contrasts are independent comparisons within each group, assessing differences between specific repetitions (1–2, 1–3 and 2–3). Additionally, a Spearman correlation matrix was created between self-assessment scores and VR-OTS performance variables.

All statistic calculations and plots were created in RStudio (Version 2023.09.1 Build 494, © 2009–2023 Posit Software, PBC). The following packages were applied: for data rearrangement, Tidyverse package (Version 2.0.0); for the models and post hoc calculations, lme4 (Version 1.1-37), lmerTest (Version 3.1-3), emmeans (Version 1.11.2) and performance (Version 0.15.0); and for plotting, ggeffects (Version 2.3.0), gtsummary (Version 2.3.0) and ggplot2 (Version 2.5.3, part of Tidyverse) were used.

## 3. Results

Demographics of the groups and questionnaire scores are displayed in Table 1. All statistical tests between groups were calculated taking into account the differences in age and sex. Age was a significant covariate in RT analysis (*p*-values < 0.001), while gender showed no effect on RT values (*p* > 0.05).

**Figure 2:**
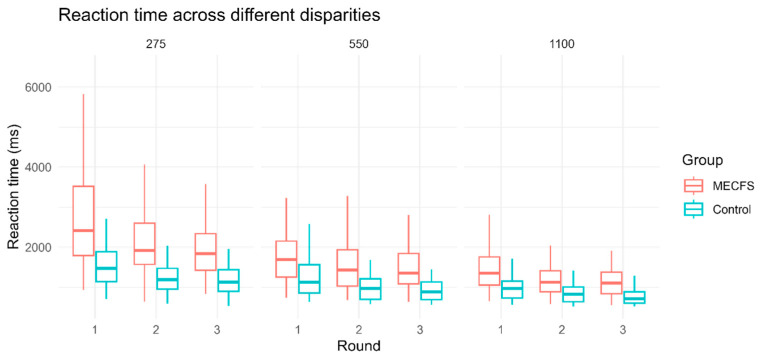
Reaction times (ms) per group (ME/CFS, control) and round (1–3) across the different disparities (275″, 550″ and 1100″). Reaction times decrease with easier disparity; patients with ME/CFS (red) perform considerably slower across all disparities and rounds.

Overall, 85% (51/60) of the controls and 85% (51/60) of patients with ME/CFS achieved a successful binocular fusion. Figure 2 shows the RTs per round for each group before accounting for the differences in age. As age was a significant covariate and controls and ME/CFS patients were not age-matched, statistical analysis of RT and RT metrics was calculated while accounting for age differences.

**Figure 3:**
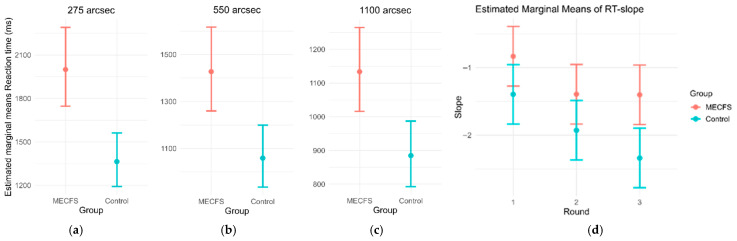
(**a**–**c**) Estimated means of reaction times per group and disparity across all rounds. Accounting for age and sex, all results remain statistically significant (**a**) at disparity 275: 1969 ms vs. 1384 ms (*p* = 0.0001); (**b**) at disparity 550: 1409 vs. 1071 ms (*p* = 0.0012); and (**c**) at disparity 1100: 1126 ms vs. 891 ms (*p* = 0.00223). (**d**) Estimated means of RT-slope per round, accounting for age and sex. RT-slope references the slope between the individuals’ reaction speed from hardest to easiest stimulus. This slope steepens when reaction times are optimized. In rounds 1 and 2, there is no statistically significant difference between groups, although ME/CFS RT-slopes were less steep on average (R1 *p* = 0.0779, R2 *p* = 0.1010). In round 3, a statistical difference between groups can be shown (R3 *p* = 0.0042), as the RT-slope of controls improved further, while RT-slope of ME/CFS patients stayed similar to R2. When comparing RT-slope within groups from R1 to R3, controls improved significantly (0.92 units (95% CI: 0.3 to 1.2), *p* = 0.0015), while ME/CFS only showed a trend of improvement (0.56 units (95% CI: −0.05 to 1.18), *p* = 0.0799).

When accounting for the covariate of age and analyzing the estimated marginal mean RT across all game rounds, ME/CFS patients lagged significantly behind the controls: 1969 ms vs. 1384 ms (275″; *p* = 0.0001), 1409 vs. 1071 ms (550″; *p* = 0.0012) and 1126 ms vs. 891 ms (1100″; *p* = 0.00223) (Figure 3a–c). In order to better understand the RT dynamics across all disparities per game round, individuals’ RT-slope is calculated by the decrease in RT from the more difficult stimuli to the easier stimuli within each round (Figure 3d). ME/CFS patients’ RT-slopes were less steep on average, which was not statistically significant during the first two rounds (R1 *p* = 0.0779, R2 *p* = 0.1010), but this difference nearly doubled during round 3 compared to previous rounds (*p* = 0.0042). Alongside this, the RT-slope of controls improved significantly from round 1 to round 3 (0.92 units (95% CI: 0.3 to 1.2), *p* = 0.0015), while the ME/CFS cohort showed only a trend of improvement (0.56 units (95% CI: −0.05 to 1.18), *p* = 0.0799) (Figure 3d).

As these results suggest altered RT dynamics relative to stimulus difficulty with a significant drop in performance by round 3, the RT change between disparities was analyzed. Both groups improved their RT by round 2 (all pairwise contrasts *p* < 0.0001); however, by round 3 this improvement was diminished in the MECFS group compared to controls. Table 2 displays the changes in RT during gaming.

**Figure 4:**
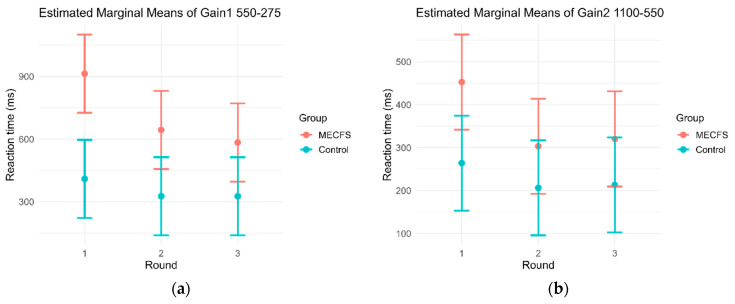
EMM plots of the increase in RT from the easier to the harder disparity (550″-275″ and 1100″-550″). This metric reports within an individual round the impact of visual cognition time on the total RT. (**a**) GAIN1 (delta 550″-275″) was significantly higher in ME/CFS patients in R1 and R2 compared to controls (R1 *p* = 0.0003, R2 *p* = 0.00295) and improved significantly from R1 to R2 (*p* = 0.0499). Gain of controls did not change across rounds (*p* > 0.05). (**b**) GAIN2 showed a similar pattern to GAIN1, with ME/CFS patients performing significantly worse in R1 (*p* = 0.0462) and showing a trend of improvement in R2 (*p* = 0.575). The difference between both groups at these easier disparities was not as pronounced.

**Figure 5:**
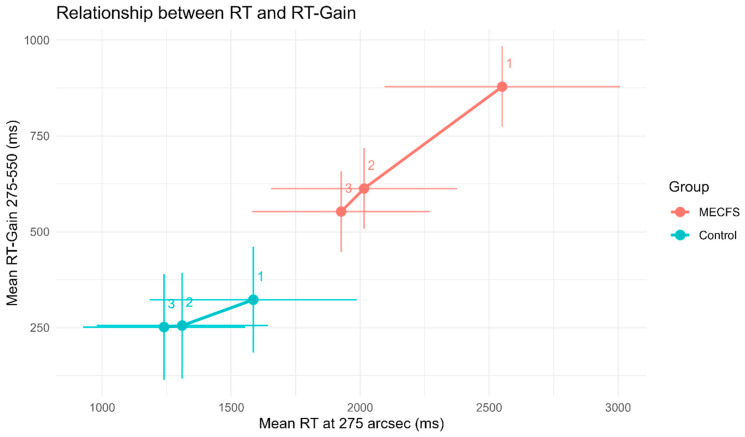
The relationship between RT at 275″ and RT gain from 275″ to 550″ disparities within a round. Dots represent the estimated means after considering age and gender, with error bars illustrating standard deviations, and are labeled by round. Lines are flatter if cognition had less of an impact on RT improvement between rounds. ME/CFS patients start out considerably slower, and cognition played a larger role in improving their reaction speed between rounds.

The differences in RT of adjacent disparities were a further point of interest. VR-OTS defined GAIN1 to be the gain of RT between 550″ and 275″ disparities, and GAIN2 between 1100″ and 550″ disparities. RT comprises two big factors, the visual–neurocognitive reaction time it takes to decide which stimulus is the correct one, which is subject to change according to the difficulty, and the motoric reaction time, which should be roughly the same within an individual, regardless of how difficult the decision process was before [24]. Interestingly, GAIN1 and GAIN2 remained nearly constant during the three game rounds in controls, while they significantly improved from R1 to R2 in the ME/CFS cohort. The difference in GAIN between controls and ME/CFS patients was most pronounced during R1 (GAIN1: 492.84 ms (95% CI: 227.92 ms to 757.76 ms), *p* = 0.0003; GAIN2: 156.16 ms (95% CI: 2.67 ms to 309.65 ms), *p* = 0.0462), Figure 4). The relationship between RT at 275″ and the improvement in RT between 550″ and 275″ (GAIN1) shows how much cognition impacts RT improvement in each round. This is illustrated in Figure 5. If a participant increases their RT between rounds but has no change in GAIN1, the improvements can be assumed to be largely due to better motoric handling of the device controls. ME/CFS patients are much slower in R1, and GAIN1 impacted their RT improvement in R2 and R3, while controls performed markedly better, and their RT improvements were largely motoric.

### Self-Assessment Correlation

**Figure 6:**
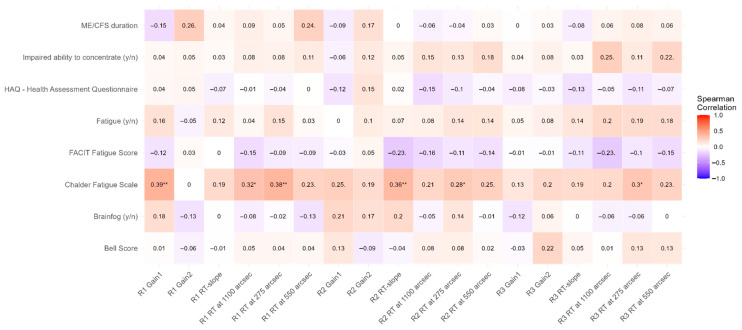
Spearman correlation plot between self-reported fatigue, assessed by fatigue scores and VR-OTS RT performance metrics. Dots (. for *p* < 0.1) and stars (* for *p* <0.05 and ** for *p* <0.01) after numbers reference trends and significance. Overall self-assessments did not correlate with VR-OTS performance. Only the Chalder Fatigue Scale had weak to moderate correlations with RT at 275″ disparity stimuli in all rounds. The largest correlation was calculated between the Chalder Fatigue Scale and R1 RT at 275″, r = 0.38, *p* = 0.0027.

ME/CFS severity and treatment success are commonly graded on self-assessment questionnaires. A Spearman correlation matrix was created to assess whether the RT metrics of ME/CFS patients correlated with their self-reported fatigue (assessed by Bell Score, FACIT Fatigue Score, Chalder Fatigue Scale, and HAQ), the binary anamnestic questions (brain fog (yes/no), fatigue (yes/no), and impaired concentration (yes/no)) or the disease duration (Figure 6). Only the Chalder Fatigue Scale had significant weak to moderate correlations with RT at 275″ disparities in all rounds (largest correlation with R1 RT at 275″, r = 0.38, *p* = 0.0027), among other sporadic correlations which were not present in all game rounds.

## 4. Discussion

Cardinal diagnostic criteria for ME/CFS in the international consensus are based on self-reported rapid fatigability, difficulty processing information and motor- and neurosensory perceptual disturbances including impaired depth perception [10]. As such, a complex reduction in cognitive performance in ME/CFS has been well demonstrated, with some tests showing slower processing speeds in the ME/CFS group compared to controls, with the same performance accuracy [29]. Functional MRI studies support these findings, with observations of additional brain area recruitment and slower signal responses [6]. VR-OTS analyzes oculomotor function, visual reaction and visual processing speed in an easy-to-understand setting of a 3D game. We recently established VR-OTS as an assessment in patients with PCS [23,24]. In PCS, RT is reduced compared to controls [23]. Kelly et al. reported similar results for a combined test set of oculomotor, vestibular, RT and cognitive tests in PCS [30]. Therefore, we hypothesized that VR-OTS may be a useful tool in objectifying cognitive impairments in ME/CFS patients. Table 3 compares the previous studies cited in this work to the current study. As the impact and existence of ME/CFS is still subject of polarizing opinions [31], objective tools may aid in diagnosis, disease awareness and understanding in both physicians and the general population.

### 4.1. RT Slowing in ME/CFS

ME/CFS patients had considerably different RT patterns when compared to healthy controls. They were slower in every round and every disparity, and while all participants improved their speed from the first round to the third, the difference between groups became more pronounced by game 3, particularly at the easiest disparity (1100″). Here, controls were able to significantly improve their RT once more, but ME/CFS patients did not. This may be a result of fatigue, as by this point, full attention had already been required for an extended period of time. When comparing the RT improvement between groups in Table 2, a greater percentage improvement in the ME/CFS group from round 1 to round 2 can be observed. Rather than this being a sign of “better learning” in the ME/CFS group, we consider this to be a circumstance of the considerably slower RT in the ME/CFS group, especially since pairwise comparisons in round 2 still show that the RT of controls is much faster (39.7% (15.8% to 68.5%, *p* = 0.0005)). Similarly, when the change from round 2 to round 3 at the most difficult disparity 275″ is observed, both groups appear to improve by about the same fraction (ME/CFS: −4.6% (+3.4% to −13.2%, ns) vs. control −5.7% (+2.4% to −14.3%, ns)). Round-to-round improvements can only be properly considered in the context of within-group effects, as this effect is non-linear and limited by physiological factors as RT cannot be 0 or lower. Hence, it is more difficult for the control group to improve their RT, particularly at harder difficulties, as they had already started at a significantly faster RT. This is likely also the reason why a significant improvement in controls from round 2 to round 3 can only be seen at the easiest difficulty.

This circumstance is somewhat negated when analyzing the RT-slope. This parameter is the slope of the best-fit line of the RT of each disparity when ordered most difficult to easiest within one round. As the ease of differentiation between disparities is non-linear, the performance at easier disparity levels should improve disproportionally faster, resulting in a steeper slope by subsequent rounds. This effect is indeed visible in the control group from round to round but is greatly ameliorated in the ME/CS group from round 1 to round 2 and fully absent from round 2 to round 3 (Figure 3d). This means that something other than the stimulus difficulty alone impairs RT in ME/CFS. It is our hypothesis that this effect may be a reflection of neurotrophic effects of the disease. While ME/CFS patients may have some underlying motivations to simulate or overstate their impairments in order to receive recognition or benefits, we do not believe this to be a concern in the present study. Firstly, this was a voluntary study, and participants did not gain reports of their individual performance. Therefore, the performance could not be used as ‘proof’ of impairment to receive insurance or retirement benefits. Secondly, the pattern of our results does not support intentional RT slowing: Figure 2 shows box-and-whisker plots of the performance of the ME/CFS group and controls across each round and difficulty without any adjustment for age or other factors. These are proportional to difficulty within rounds, and the mean difference between groups across all rounds ranges from 235 ms at the easiest difficulty to 585 ms at the hardest difficulty when accounting for age (Figure 3a,c). During each round, the three difficulties are shown at random; hence, it would be very difficult to simulate a slowed RT which is proportional to difficulty and less than a second slower than healthy controls. Additionally, the participants performed the test alone and for the first time, so participants had no prior knowledge of how long a “normal” RT is. In further studies, when the device is tested for repeated test viability and use in diagnostic support, the proportion of RT between difficulties within a round will be used to assess for test validity.

### 4.2. Dynamic of RT Improvements Across Rounds

As ME/CFS patients suffer both neurocognitive and neuro-/muscular impairments, a further point of interest was whether the decreased RT was mainly caused by delays in visual–cognitive reaction time or impaired motoric reaction time. The RT gain variables give information on which of these parameters is more likely impaired by measuring the difference between the RT from the harder and the easier stimulus RT. The resulting data reflects how much longer the subjects took (according to the visual difficulty of the stimulus), since pressing the button is assumed to take the same amount of time on average in each round. Thus, RT gain is a measure cognitive speed, independent of motoric ability. Among the control group, neither GAIN1 nor GAIN2 changed significantly across game rounds. Therefore, the RT improvement across rounds can be assumed to be mainly due to better neuromuscular coordination, i.e., learning the game controls. Contrarywise, ME/CFS patients show significant improvement from round 1 to round 2 in GAIN1 (*p* = 0.0499) and a strong trend of improvement in GAIN2 (difference: 156.26 ms (95% CI: −3.85 to 316.37), *p* = 0.0575). This suggests that ME/CFS patients possibly had greater visual–cognitive difficulty and/or understood the instructions less, thus resulting in worse performance in the first round of the game with improvement by round 2. The impact of this is illustrated in Figure 5, where the ME/CFS group does not approach the performance of controls even by round 3. While motivation and comments about fatigue were not assessed and participants’ comments were not recorded, similarly to the discussion of overall slower RT in ME/CFS above, we do not expect a disproportional decrease in motivation in either group, and the data reflects proportional population-wide changes with the standard deviations remaining similar (as represented by the error bars in Figure 5). Another explanation may be that RT in the first round is disproportionately impaired due to the relatively older ME/CFS population being less comfortable with the VR setup. Augmented reality may improve potential simulator sickness but may introduce other confounders such as distractions by other items or people in the examination room or differences in contrast due to lighting changes. The relatively sterile environment and constant lighting, which the VR setup enables, may be tied to fewer confounders considering the assessment takes place in a relatively stationary environment with fixed distances. The balls only move within the space if the participant moves their head to ensure the correct placement within the field of view is maintained. Medical trials comparing augmented and virtual reality while presenting the same test are relatively rare. Shahnewaz Ferdous et al. presented no significant difference in simulator sickness scores in a study with a similarly simple test environment between augmented and virtual reality [32]. Future studies should incorporate standardized simulator sickness assessment (e.g., SSQ with baseline correction) to exclude potential confounding effects of VR intolerance in ME/CFS.

### 4.3. Age Limitations

Unfortunately, we were unable to recruit age-matched healthy volunteers for this study. This represents a considerable limitation of the study. Although all analyses presented here were normalized for age, this modeling assumed a linear change with age. It is known that within the older population, the keenness to adopt new technologies is reduced [33] and might be associated with impaired digital reading performance [34]. Furthermore, Wan et al. [35] supposes that short-term memory predicts the success at adopting higher-level technologies. In addition to age, working memory is known to be reduced in ME/CFS [29]. Therefore, the assumption of a linear change with a singular ‘age’ factor may be wrong. In order to assess the stability of our results, an age-restricted cohort of participants between 30 and 55 years of age was selected, which yielded about half of ME/CFS participants (demographics in Table A1 of Appendix A). The same analysis calculations were performed for disparity 275″ as in Table 2 to show RT change across rounds with very similar results (Table A2 of Appendix A).

### 4.4. Lack of Correlation with Questionnaires

We had hoped to be able to show correlation between the RT metrics in VR-OTS performance and the self-assessment questionnaires (patient-reported outcome measures, PROMs). Neither anamnestic reports nor PROMs correlated with any RT outcome metrics. The greatest correlation was achieved between the Chalder Fatigue Scale and the RT at 275″ disparity. Other RT metrics showed singular correlations with the Chalder Fatigue Scale without showing a clear pattern. Rather than supposing that the predictive value of VR-OTS for ME/CFS severity is low, we assume that although these questionnaires are commonly used in diagnostics of ME/CFS and help in observing changes within a person, they are subjective. Difficulties comparing greater populations have been documented in other studies as well [36,37], particularly when the patient’s own interpretation of each question is allowed in self-assessment. This is supported by the literature, as ceiling effects and bad internal consistency [36,37] as well as poor prediction across subscales has led to PROMs being criticized even beyond scales used in ME/CFS [38,39]. Comparison of questionnaire assessments and sensor measurements for time spent upright showed not only overestimation of actual time spent upright by the ME/CFS cohort but also demonstrated the lack of correlation between PROMs and time spent upright in ME/CFS cohorts [40]. In line with this, Stussman et al. [13] recently showed that qualitative interviews are more sensitive than questionnaires when quantifying PEM changes.

### 4.5. Limitations

In addition to the limitations regarding age and possible VR intolerance as confounding factors already discussed above, the present study is limited by a lack of specificity evaluation and cannot establish a cause-and-effect relationship between RT metrics and ME/CFS. There are other causes of fatigue and possible RT deficits, such as depression, sleep deficits and other debilitating diseases which may increase RT. Among these, VR-OTS studies have been performed in PCS, which also showed increased RT in PCS participants compared to healthy controls [23]. Additionally, those who have previously trained their reaction times such as gamers and reaction-based physical sport players may have individual impairments but still present above average when compared to age-matched peers. In the current study, we failed to assess whether participants engage in activities that train RT. We further do not propose VR-OTS as a specific diagnostic test for ME/CFS but rather as a biomarker helping to quantify fatigue. Other cross-sectional studies such as testing RT before and after night shifts in healthy hospital workers have been proposed. However, VR-OTS RT metrics are not entirely unspecific: a previous study showed that VR-OTS could be used as a concussion marker by showing impaired binocular fusion capacity after head trauma [22]. Both in PCS and the present study, no significant difference in the binocular fusion capacity was found [23], but RT was slower in affected participants. Future studies aim to make better use of the data generated through the VR system and have the goal of analyzing and extracting more technical data such as pupillary movements, saccades and the time from fixation of the correct stimulus to pressing the related keyboard button. Further, as laboratory biomarkers such as mitochondrial dysfunction due to viral reactivation [8], lipid and energy metabolites [41], cerebrospinal fluid metabolites [42] and neuroimaging tools [43] have recently been identified as potential marker for PEM and fatigue, it would be interesting to correlate these markers with VR-OTS in order to identify whether a connection between other biomarkers and RT performance exists. A larger-scale study has recently begun with the aim to correlate biomarkers with performance. This identification and validation of objective biomarkers may help in diagnosis, disease awareness and understanding [31].

## 5. Conclusions

Reduced overall cognitive processing speed is well documented for ME/CFS. The relation to PEM and fatigue is harder to elucidate. VR-OTS RT metrics showed not only considerable differences between healthy controls and patients with ME/CFS, but also different performance patterns over time; thus, some of these metrics may be indicative of PEM and fatigue. As barely any correlation could be found between RT metrics and symptom severity in PROMs, further studies are recommended in order to gauge the diagnostic power of this assessment method.

## Figures and Tables

**Table 1:** Demographics and questionnaire scores of patients with ME/CFS and controls, respectively.

Characteristics	ME/CFS N = 60 ^1^	Control N = 60 ^1^	*p*-Value ^2^
Age (years)	47 (38, 58)	29 (24, 56)	0.007
Sex			0.6
Male (number)	27 (45%)	30 (50%)	
Female (number)	33 (55%)	30 (50%)	
Duration of ME/CFS (days)	3232 (2051, 6592)	NA	
Bell Score	30 (20, 30)	100 (100, 100)	<0.001
Chalder Fatigue Scale	11 (9, 11)	0 (0, 1)	<0.001
FACIT ^3^	16 (11, 23)	NA	
HAQ ^4^	1.00 (0.63, 1.63)	NA	
Brain fog (y/n)	45 (75%)	0 (0%)	<0.001
Reduced ability to concentrate (y/n)	46 (77%)	0 (0%)	<0.001
PEM ^5^/Fatigue (y/n)	52 (87%)	0 (0%)	<0.001
Binocular fusion	51/60	51/60	

^1^ Median (Q1, Q3); n (%). ^2^ Wilcoxon rank sum test; Pearson’s Chi-squared test. ^3^ FACIT—Functional Assessment of Chronic Illness Therapy Fatigue Score. ^4^ HAQ—Health Assessment Questionnaire. ^5^ PEM—post-exertional malaise.

**Table 2:** Shows the improvement in reaction times (RTs) in percentage change from round to round for each stimulus disparity and pairwise contrasts comparing the groups with each other.

	ME/CFS	*p*-Value	Control	*p*-Value
RT change from round 1 to round 2				
Disparity 275″	−26.6% (−16.9% to −37.0%)	<0.0001	−21.1% (−11.8% to −31.0%)	<0.0001
	Pairwise contrast between groups R2: 39.7% (15.8% to 68.5%)			0.0005
Disparity 550″	−20.7% (−12.6% to −29.4%)	<0.0001	−19.6% (−11.6% to −28.2%)	<0.0001
	Pairwise contrast between groups R2: 29.1% (8.6% to 53.5%)			0.0038
Disparity 1100″	−20.0% (−13.0% to −27.3%)	<0.0001	−17.3% (−10.5% to −24.4%)	<0.0001
	Pairwise contrast between groups R2: 23.5% (5.7% to 44.4%)			0.0081
RT change from round 2 to round 3				
Disparity 275″	−4.6% (+3.4% to −13.2%)	0.3766	−5.7% (+2.4% to −14.3%)	0.2326
	Pairwise contrast between groups R3: 41.1% (16.9% to 70.2%)			0.0003
Disparity 550″	−1.5% (+5.3% to −8.8%)	0.8672	−6.4% (+0.7% to −14.1%)	0.0874
	Pairwise contrast between groups R3: 35.4% (13.8% to 61.0%)			0.0006
Disparity 1100″	−2.9% (+3.1% to −9.2%)	0.5022	−7.8% (−1.6% to −14.4%)	0.0085
	Pairwise contrast between groups R3: 29.4% (10.7% to 51.3%)			0.0012

**Table 3:** Comparison of the methods and findings of cited works with the current study.

Author	Disease	Article/ Review	Key Finding	Type of Finding and Method	Limitation as ME/CFS Tool	Compared to Current Study
Scherbakov et al. [1]	ME/CFS	Article	Peripheral endothelial dysfunction	No direct functional correlation	No functional information	N/A
Clarke et al. [2]	ME/CFS	Review	Review of potential blood biomarkers	No direct functional correlation	No functional information	N/A
Freitag et al. [3]	ME/CFS	article	Autoantibodies to vasoactive G-PCR correlate with symptom severity	Correlation with fatigue symptoms (PROM)	No functional information	N/A
Bizjak et al. [4]	ME/CFS and PCS	Article	Mitochondrial differences	Functional with exercise testing	Requires exercise testing, which may trigger PEM	Study focusses on mitochondrial function and compares with exercise testing. The focus is on physical performance, rather than neurological.
Shan et al. [6]	ME/CFS	Review	Review of neuroimaging characteristics including fMRI	Functional with fMRI	Requires fMRI access	Shows slower signal responses and altered brain area recruitment in fMRI studies, supporting the findings of the current study.
Lange et al. [29]	ME/CFS	Article	Slower processing speed across timepoints, independent of exercise test on same visit	Traditional tests and CogState clinical functional assessment tests (computerized cognitive assessment) with tasks reflecting psychomotor speed, attention, learning memory, and learning efficiency	Multiple separate tests administered once at beginning and end of a clinic visit and at home after clinic visits on conventional devices. Although tasks are said to reflect multiple facets of cognition, response time is the only outcome reported as significant.	The findings support the current study; rather than three successive administrations in the current study, multiple tasks were administered over a larger period of time. Additionally, three-dimensional tasks are reported to impose greater cognitive loads [21].
Scribano et al. [17]	Alzheimer’s disease	Review	Review of VR and AI methods to detect preclinical Alzheimer’s disease	Some functional markers using VR and AI algorithms	N/A	Reviews functional testing using VR in the detection of preclinical Alzheimer’s disease.
Culicetto et al. [18]	Parkinson’s disease	Review	Review of VR/eye tracking as diagnostic and disease markers	Functional with VR glasses and other eye trackers	N/A	Reviews functional testing using VR as diagnostic marker of Parkinson’s disease.
Faúndez et al. [19]	Persistent postural-perceptual dizziness	Article	Eye tracking and VR spatial navigation test shows deficits in affected patients	Functional with VR glasses and normal screens (compared)	N/A	Tests spatial cognition in persistent postural-perceptual dizziness and vestibular disorders.
Kara et al. [22]	Mild traumatic brain injury	Article	Fusion capacity is reduced in mild traumatic brain injury	Functional with VR-OTS	N/A	Same test, in contrast to mild traumatic brain injury, where binocular fusion capacity was reduced. ME/CFS patients had the same rate of fusion as controls.
Güttes et al. [23]	PCS	Article	Reaction time deficits	Functional with VR-OTS	N/A	Same test-similar to the current study PCS patients had slower RT compared to controls.
Kelly et al. [30]	PCS	Article	VR tests showed neural deficits	Functional with 14 tests on a VR eye-tracking device targeting oculomotor, vestibular, reaction time and cognitive tests	N/A	This study supports the findings of slower RT also reported in our test; although their discussion draws links to ME/CFS, this is not supported by data.

## Data Availability

The dataset analyzed during the current study is not publicly available due to general data privacy regulations but is available from the corresponding authors.

## Abbreviations

The following abbreviations are used in this manuscript:

ME/CFS	Myalgic encephalomyelitis/chronic fatigue syndrome
PEM	Post-exertional malaise
RT	Reaction time
EM-means	Estimated marginal means
PCS	Post-COVID syndrome
2D	Two-dimensional
3D	Three-dimensional
VR-OTS	Virtual reality–oculomotor test system
HAQ-DI	Health Assessment Questionnaire Disability Index
FACIT	Functional Assessment of Chronic Illness Therapy
SE	Standard error
CI	Confidence interval
PROM	Patient-reported outcome measures

## Appendix A

An age-restricted sub-cohort of participants between 30 and 55 years of age was calculated to show stability of results.

**Table A1:** Demographics of the age-restricted subset.

Characteristic	ME/CFS N = 29 ^1^	Control N = 13 ^1^	*p*-Value ^2^
Age (years)	43 (39, 46)	48 (44, 52)	0.12
Sex			0.4
Male (number)	14 (48%)	8 (62%)	
Female (number)	15 (52%)	5 (38%)	

^1^ Median (Q1, Q3); n (%). ^2^ Wilcoxon rank sum test; Pearson’s Chi-squared test.

**Table A2:** Comparison of RT change at 275″ (the hardest disparity with the least change within groups from round 2 to round 3) between the subset and whole cohort.

	ME/CFS	*p*-Value	Control	*p*-Value
RT change from round 1 to round 2				
Whole cohort 275″	−26.6% (−16.9% to −37.0%)	<0.0001	−21.1% (−11.8% to −31.0%)	<0.0001
	Pairwise contrast between groups R2: 39.7% (15.8% to 68.5%%)			0.0005
Subset 275″	−35.3% (−21.1% to −51.2%)	<0.0001	−15.3% (+2.2% to −36.0%)	0.105
	Pairwise contrast between groups R2: 61.5% (16.2% to 124.6%)			0.0044
RT change from round 2 to round 3				
Whole cohort 275″	−4.6% (+3.4% to −13.2%)	0.3766	−5.7% (+2.4% to −14.3%)	0.2326
	Pairwise contrast between groups R3: 41.1% (16.9% to 70.2%)			0.0003
Subset 275″	−0.9% (+9.7% to −12.7%)	0.980	−7.6% (+8.8% to −26.8%)	0.554
	Pairwise contrast between groups R3: 72.1% (23.8% to 139.4%)			0.0012
